# Current landscape of fecal microbiota transplantation in treating depression

**DOI:** 10.3389/fimmu.2024.1416961

**Published:** 2024-06-25

**Authors:** Qi Zhang, Yajun Bi, Boyu Zhang, Qiong Jiang, Chao Kam Mou, Lelin Lei, Yibo Deng, Yutong Li, Jing Yu, Wei Liu, Jinzhu Zhao

**Affiliations:** ^1^ Department of Plastic and Cosmetic Surgery, Tongji Hospital, Tongji Medical College, Huazhong University of Science and Technology, Wuhan, Hubei, China; ^2^ Xianning Medical College, Hubei University of Science & Technology, Xianning, Hubei, China; ^3^ Department of Pediatrics, Dalian Municipal Women and Children’s Medical Center (Group), Dalian Medical University, Dalian, Liaoning, China; ^4^ Wuhan Britain-China School, Wuhan, Hubei, China; ^5^ Department of Public Health, Tongji Hospital, Tongji Medical College, Huazhong University of Science and Technology, Wuhan, Hubei, China; ^6^ Division of Child Healthcare, Department of Pediatrics, Tongji Hospital, Tongji Medical College, Huazhong University of Science and Technology, Wuhan, Hubei, China

**Keywords:** depression, gut microbiota, fecal microbiota transplantation, gut-brain axis, gut dysbiosis, immune regulation

## Abstract

Depression, projected to be the predominant contributor to the global disease burden, is a complex condition with diverse symptoms including mood disturbances and cognitive impairments. Traditional treatments such as medication and psychotherapy often fall short, prompting the pursuit of alternative interventions. Recent research has highlighted the significant role of gut microbiota in mental health, influencing emotional and neural regulation. Fecal microbiota transplantation (FMT), the infusion of fecal matter from a healthy donor into the gut of a patient, emerges as a promising strategy to ameliorate depressive symptoms by restoring gut microbial balance. The microbial-gut-brain (MGB) axis represents a critical pathway through which to potentially rectify dysbiosis and modulate neuropsychiatric outcomes. Preclinical studies reveal that FMT can enhance neurochemicals and reduce inflammatory markers, thereby alleviating depressive behaviors. Moreover, FMT has shown promise in clinical settings, improving gastrointestinal symptoms and overall quality of life in patients with depression. The review highlights the role of the gut-brain axis in depression and the need for further research to validate the long-term safety and efficacy of FMT, identify specific therapeutic microbial strains, and develop targeted microbial modulation strategies. Advancing our understanding of FMT could revolutionize depression treatment, shifting the paradigm toward microbiome-targeting therapies.

## 1 Introduction

Depression poses a significant public health challenge worldwide, not only due to its impact on individuals but also as a primary catalyst for suicide (1, 2). The World Health Organization forecasts that by 2030, depression is expected to become the leading contributor to the global disease burden (3). Depression, once merely categorized as an emotional disturbance, is now acknowledged as a complex disorder characterized by a spectrum of emotional, physical, and cognitive symptoms (4, 5). Depression can lead to cognitive impairment, reflecting a significant impact on mental processing and functioning (6, 7). Manifestations of depression include insomnia or hypersomnia, persistent fatigue, loss of appetite, and mood fluctuations, with severe cases posing a potential threat to life (8).

Contemporary studies have elucidated that the gut microbiota, an intricate ecosystem consisting of bacteria, viruses, archaea, and fungi, is integral to the sustenance of human health (9). The gut microbiota influences individual emotional equilibrium by regulating neural circuits and modulating the release of neurotransmitters within the central nervous system, thus providing novel insights into the biological foundations of mood disorders. Fecal microbiota transplantation (FMT), a technique for reconstituting the gut microbiota, has demonstrated promising therapeutic potential for ameliorating depression in preclinical studies (10). This procedure involves the transfer of fecal matter from a healthy donor into the gastrointestinal tract of a patient, aiming to restore a balanced microbial ecosystem. For example, Cai et al. found that rats subjected to chronic unpredictable mild stress (CUMS) and treated with FMT showed elevated hippocampal levels of neurochemicals like 5-HT, gamma-aminobutyric acid (GABA), and brain-derived neurotrophic factor (BDNF), coupled with reduced inflammatory markers, leading to an alleviation of depressive symptoms (11). Similarly, Hu et al. observed that rats with depressive-like behaviors experienced significant improvement after receiving gut microbiota from healthy donors, highlighting FMT capacity to influence mood regulation (12).

This review concentrates on contemporary studies examining the influence of gut microbiota on depression and delves into the potential and underlying mechanisms of FMT as a novel therapeutic strategy for mitigating depressive symptoms. These insights not only emphasize the biological foundations of depression but also reveal the pivotal importance of the gut microbiome in mental health remodeling and therapy.

## 2 Overview of the connection between gut microbiota and depression

### 2.1 Gut microbiota microenvironment

The gastrointestinal tract is commonly referred to as the second brain due to its critical roles in digestion, immune response, and endocrine regulation (13, 14). Within the gut of a healthy adult resides a vast and diverse array of microorganisms, including bacteria, archaea, micro-eukaryotes, fungi, and viruses, collectively forming the gut microbiota (15). The composition of the gut microbiota exhibits life-stage-specific dynamics, with fluctuations during childhood, a period of relative stability during adulthood, and subsequent shifts associated with aging (16). Despite its susceptibility to genetic and environmental factors, including diet, stress, and antibiotics (ABX) exposure, the gut microbiota remains adaptable, performing essential metabolic and biochemical functions vital for host homeostasis (17).

The gut microenvironment primarily fosters the proliferation of bacterial phyla, such as *Firmicutes*, *Bacteroidetes*, *Actinobacteria*, *Fusobacteria*, *Proteobacteria*, *Verrucomicrobia*, and *Cyanobacteria* (18). Gut dysbiosis, characterized by disturbances in the microbiota composition and quantity, has been linked to a host of disorders, including gastrointestinal motility issues, malabsorption, and mental health conditions (19, 20). This dysbiosis is implicated in the pathophysiology of depression. Comparative analyses reveal that while *Firmicutes* and *Bacteroidetes* dominate the fecal microbiota of healthy individuals, depressed individuals exhibit significant alterations, including decreased *Lachnospiraceae* and *Ruminococcaceae*, along with reduced populations of *Fecalibacterium* and *Ruminococcus*, and lower levels of *Lactobacillus* and *Bifidobacterium* (21).

### 2.2 Evidence of gut microbiota in inducing depression

**Figure 1:**
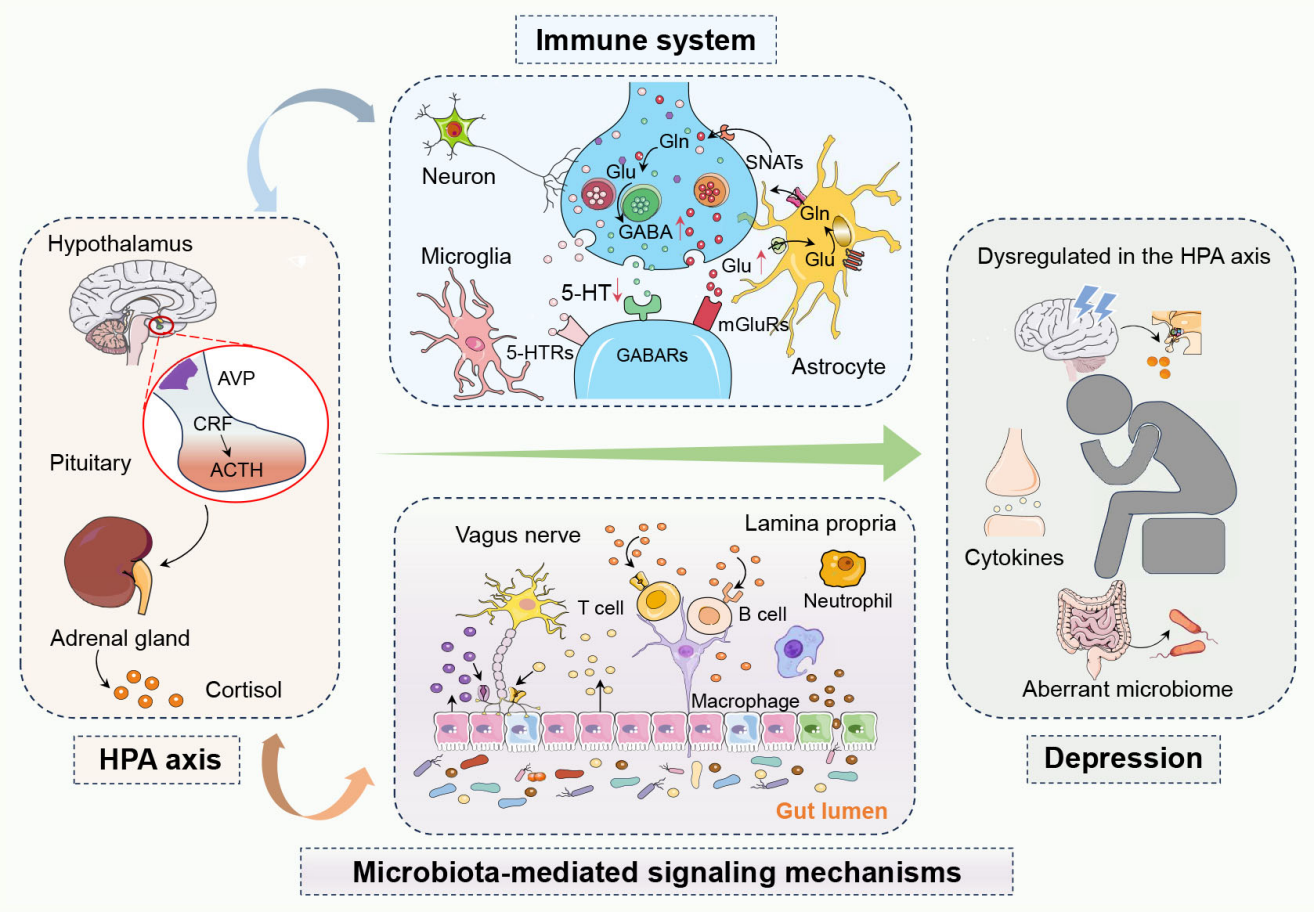
The complex network of interactions between the hypothalamic-pituitary-adrenal (HPA) axis, the immune system, and microbiota-mediated signaling mechanisms in depression. In depression, dysregulation of the hypothalamic-pituitary-adrenal (HPA) axis leads to abnormal cortisol levels, while an overactive immune system causes elevated inflammatory markers. The gut microbiota influences brain function and mood regulation both directly and indirectly through the gut-brain axis and metabolic byproducts like short-chain fatty acids. Activation of the HPA axis can trigger immune responses and inflammation, creating a vicious cycle where inflammation further affects the HPA axis. Changes in the gut microbiota can also impact emotion and behavior by affecting the functionality of these systems, playing a critical role in the development of depression. In summary, these systems interact with each other, forming a complex pathophysiological network in depression. 5-Hydroxytryptamine, 5-HT; 5-Hydroxytryptamine receptors, 5-HTRs; Adrenocorticotropic hormone, ACTH; Arginine vasopressin, AVP; Corticotropin-releasing factor, CRF; Gamma-aminobutyric acid, GABA; Gamma-aminobutyric acid receptors, GABAs; Glutamine, Gln; Glutamate, Glu; Hypothalamic-pituitary-adrenal, HPA; Metabotropic glutamate receptors, mGluRs; Sodium-coupled neutral amino acid transporters, SNATs.

Transferring the fecal microbiota from patients with major depressive disorder (MDD) into rodents has been shown to induce depression-like behaviors, suggesting the influence of gut microbiota on emotional states (22). Notably, there are marked differences between the microbiota of FMT-MDD and FMT-Healthy groups (23). These findings suggest that microbial dysbiosis may not only be associated with but could potentially precede and contribute to the onset of depression. The complex and dynamic nature of the gut microbiota has significant implications for mental health, particularly depression (24). The interplay between microbiota composition and host factors underscores the need for a deeper understanding of gut-brain axis mechanisms (**Figure 1**).

#### 2.2.1 Gut microbiota in inducing depression via block coprophagy

Coprophagy, the ingestion of feces from the same or different species, allows rodents to preserve essential gut microbiota diversity and function through this scavenging behavior (25, 26). Sha et al. discovered that blocking coprophagy in healthy mice led to increased levels of depression and pro-inflammatory cytokines (27). Furthermore, when the fecal microbiota of mice with CUMS mice and lipopolysaccharide (LPS) mice were transplanted into healthy recipient mice, the coprophagy-blocked group exhibited more severe depressive symptoms and higher levels of pro-inflammatory cytokines, in the serum, prefrontal cortex (PFC), and hippocampus, compared with the coprophagy-unblocked group. Thus, autophagy inhibition appeared to amplify inflammatory responses and precipitate depressive behaviors in both normal mice and those receiving FMT from disease model donors.

#### 2.2.2 Gut microbiota from patients with rheumatoid arthritis in inducing depression

Rheumatoid arthritis (RA) and depression are prevalent diseases that harm patient quality of life and impose a significant economic burden on society (28, 29). Depression is a frequent comorbidity in patients with RA, which not only reduces treatment effectiveness but also increases the risk of disability and death (30). Moreover, there appears to be a bidirectional link between depression and RA (31). Pu et al. examined the impact of FMT from RA patients on depression-like behaviors using the mouse model of collagen-induced arthritis (32). Before FMT, mice underwent ABX treatment to deplete their endogenous gut microbiota. FMT from patients with RA into the ABX-treated mice resulted in depression-like behavior, changed gut microbiota composition, elevated levels of IL-6 and TNF-α, and downregulated levels of synaptic proteins in the PFC. In addition, significant correlations were observed between the relative abundance of microbiota and plasma cytokines, expression of synaptic proteins in the PFC, or depression-like behavior. In the RA FMT group, the ratio of Peyer’s patches and splenic CD4^+^ T cells to Th1/Th2 increased, while the ratio of Treg cells decreased. These findings suggest that FMT from RA patients induced depressive-like behaviors in ABX-treated mice through T cell differentiation, providing evidence for the involvement of the gut-microbiome-brain axis in depression.

#### 2.2.3 Stimuli in inducing depression via shaping gut microbiota

The emergence of depressive symptoms is often a multifactorial process where various elements such as chronic alcohol misuse, the negative side effects of certain medications, and the habitual abuse of substances can play a significant role (33, 2). These factors, individually or in combination, can lead to alterations in an individual’s emotional and psychological equilibrium, thereby potentially triggering the onset or intensifying the severity of depressive symptoms (34). Prolonged exposure to these conditions can disrupt the neurochemical balance within the brain, affecting mood-regulating neurotransmitters and leading to sustained mood disturbances (35). The complex interplay between pharmacological stimuli, psychological stressors, neurobiological alterations, and systemic changes highlights the intricate nature of depression, in which drugs such as alcohol, 5-Fluorouracil (5-FU), and methamphetamine (METH) have been identified to be tightly associated with depression.

Alcohol, commonly known as ethanol, is recognized as one of the most frequently abused substances globally (36). Recent studies indicate a positive correlation between the amount of alcohol consumption and the likelihood of developing depression (37) (38). The recognized significance of the gut microbiome has spurred research into its role in mediating neurotoxic effects associated with ethanol exposure. There was a marked distinction in the gut microbiota composition between alcoholics and healthy individuals, characterized by a significant increase in the abundance of *E. faecalis* in alcoholics (39). Zhao et al. transplanted fecal microflora from alcoholic patients into mice with gut microflora severely suppressed by ABX, demonstrating that recipients decreased BDNF, alpha 1 subunit of α1GABA_A_R in mPFC, and decreased mGluR1, PKC ϵ in NAc (40). Therefore, FMT from alcoholic patients could reduce the level of mGluR1/PKC ϵ, and induce anxiety and depressive behavior in mice. On the contrary, the FMT from mice to chronic ethanol exposure (CEE) in healthy recipient mice led to the emergence of depressive behavioral characteristics, neuroinflammatory responses, and activation of the NLRP3 inflammasome (41). Furthermore, the hippocampal downregulation of NLRP3 expression exhibited a mitigating effect on the depression-like behavioral manifestations and neuronal damage induced by CEE. Consequently, FMT produced positive treatment of CEE-induced hippocampal NLRP3-mediated neuroinflammation and depressive-like behaviors.

5-FU is a fluorinated pyrimidine analog that acts as an antimetabolite by replacing the hydrogen atom at the C-5 position of uracil with fluorine (42). This substitution facilitates the incorporation of 5-FU instead of thymine into DNA, resulting in aberrant adenine-uracil/5-FU base pairing (43). Clinically, 5-FU has been extensively utilized in the treatment of several gastrointestinal cancers, such as colorectal cancer, which is one of the most common malignancies worldwide (44, 45). Despite its broad therapeutic applications and being considered relatively safe within the spectrum of chemotherapeutic drugs, 5-FU carries a risk of specific side effects and toxicity (46, 47). The imbalance of the intestinal microbiota was commonly acknowledged to be linked with gastrointestinal lesions induced by 5-FU (48). Zhang et al. established a rat model to evaluate depression-like behaviors in 5-Fu-treated rats (48). The results demonstrated that 5-FU-induced depression-like behavior reduced the diversity of bacterial communities, altered the composition of bacterial communities, and caused changes in PFC metabolism. Furthermore, FMT from healthy donors into 5-Fu-treated rats reversed the 5-Fu-induced depression-like behaviors, restored PFC metabolism to normal levels, and alleviated amino acid imbalances in both the peripheral and central nervous systems. Therefore, 5-Fu caused depression-like behaviors through dysregulation of the microbiome-gut-brain axis, which FMT methods could reverse.

METH, commonly referred to as crystal meth, is a synthetic stimulant drug that belongs to the amphetamine class of compounds that have an exciting effect on the central nervous system, resulting in increased heart rate, increased blood pressure, increased alertness and energy, and appetite suppression (49, 50). The prolonged use of METH and its sudden withdrawal lead to substance withdrawal syndrome, encompassing symptoms such as anxiety, depression, and other manifestations. Concurrently, METH-dependent individuals experience substantial alterations in the composition of their gut microbiota, characterized by heightened alpha diversity and the relative abundance of distinct microorganisms (51). Notably, the relative abundance of *Rikenellaceae* could serve as a potential diagnostic biomarker to diagnose METH withdrawal syndrome. FMT was performed on recipient mice using fecal samples from METH addicts and METH-treated mice resulting in the induction of anxiety and depression-like behaviors in recipient mice, which could be reversed by metformin, through the regulation of microbiota-derived metabolites such as creatinine.

#### 2.2.4 Antibiotic-induced depression via shaping gut microbiota

Administering ABX markedly diminishes the fecal bacterial population and exerts a depressive impact on the microbiota composition (52). Li et al. demonstrated that after depleting the gut microbiota of mice using an ABX cocktail, FMT from CUMS-exposed mice induced anxiety-like and depressive behaviors in recipient mice, associated with changes in their gut microbiota, notably decreased lactobacillus and increased Akkermansia (53). Further research transplanted feces from chronic social defeat stress mice and control mice into ABX-treated recipient mice and discovered that the anhedonia-like phenotype observed in ABX-treated mice after FMT might be associated with two specific microorganisms, *Lactobacillus intestinalis* and *Lactobacillus reuteri* (54). And, subdiaphragmatic vagotomy significantly reversed these behavioral and biochemical abnormalities, revealing the role of the gut-brain-microbiome axis in the pathogenesis of depression via the subdiaphragmatic vagus nerve.

The specific ABX regimen precipitates depressive behaviors by altering the gut microbiota. Moreover, ABX-induced depressed mice exhibited notable differences in the abundance of gut microbiota, neurobiological factors, and functional gene abundance (55). ABX mixtures caused depression-like behavior in mice. FMT from antibiotic-induced depressed mice to normal mice resulted in the development of depression-like behavior, along with significantly reduced levels of norepinephrine, 5-HT, and BDNF in the hippocampus and PFC tissues (55). Those with ABX-induced depressive behavior exhibited reduced gut microbial diversity, activated taurine pathway, and increased abundance of functional gene lipA. Remarkably, ABX-induced depletion of donor microbiota has significant implications for the development of behavioral, biochemical, and other depressive phenotypes induced by FMT in recipient mice.

## 3 FMT in alleviating depression via microbial-gut-brain axis

The bidirectional communication between the brain and gut microbiota is achieved through multiple pathways, including the vagus nerve, neuroendocrine system, neuroimmune system, and autonomic nervous system (56, 57). The microbiota and their metabolites play a crucial role in gut-brain signaling, forming the conceptual framework of the microbial-gut-brain (MGB) axis (58). The MGB axis is considered to be associated with the onset and progression of various neuropsychiatric disorders, including depression, anxiety, and autism spectrum disorders (59). The MGB axis includes neural signal networks, immune signal networks, and chemical signal networks (60, 61). First, through the regulation of intestinal peristalsis by the autonomic nervous system, the brain’s exogenous parasympathetic and sympathetic nerves influence the activity of the internal intestinal neuron network, thereby regulating intestinal peristalsis and the rate of content transport (62). Secondly, the central efferent nerve of the brain, directly or through the enteric nervous system, is in contact with the intestinal secretory cells, regulating the secretory substances of the luminal cells, directly acting on the microbiome, and regulating microbial host signaling. In addition, the brain also affects the microbiome by regulating host immunity, maintaining the balance of the immune defense system on the surface of the intestinal mucosa, and thus affecting the composition of the microbiome. In summary, the brain-gut-microbiome axis plays an important role in regulating mood, peristalsis, and immune response, providing important insights into understanding and treating related depressive symptoms.

FMT represents an innovative therapeutic modality centered on the extraction, purification, and isolation of beneficial microbial consortia from the feces of healthy donors, followed by their transplantation into the gastrointestinal tract of recipients (63). This intervention is designed to reconstruct the gut microbiota ecosystem, thereby offering a potential treatment modality for a variety of diseases. As a microbial modulation technique, FMT has demonstrated efficacy in restoring gut microbiota after the failure of ABX therapy, effectively treating recurrent *Clostridium difficile infection*, and preventing its relapse (64).

The gut microbiota serves as a critical mediator of bidirectional gut-brain communication, potentially influencing mood regulation and cognitive behavior (65). Dysbiosis of the gut microbiota has been closely linked to the pathogenesis and progression of depression, positioning it as a novel target for therapeutic intervention (66). FMT can reverse or restore ecological imbalances by improving gut-brain axis function, potentially serving as an efficacious approach to alleviating symptoms of depression (67). Research indicates that FMT may exert positive effects on the central nervous system by modulating gut microbiota composition and activating beneficial signaling pathways within the gut-brain axis (68). Moreover, the potential of FMT to modulate immune responses, reduce inflammation levels, and enhance gut barrier function, offers a new perspective in the treatment of depression (69). The synergistic action of these mechanisms may help alleviate neuroinflammation and depressive symptoms associated with gut microbiota dysbiosis.

The vagus nerve is the primary neural conduit between the brain and the gastrointestinal tract, playing a crucial role in modulating gut activity and conveying visceral sensory signals to the brain. The vagus nerve also transmits signals from the brain, influencing gastrointestinal motility and secretion. To investigate the impact of the MGB axis on resilience, Wang et al. transplanted feces from chronic social defeat stress-susceptible mice into ABX-treated Ephx2 Knockout (KO) mice, triggering depressive-like behaviors (70). *Faecalibaculum rodentium* (*F. rodentium*) was significantly implicated in this effect. Concomitantly, there were increased IL-6 levels and diminished synaptic protein expression in the PFC. However, subdiaphragmatic vagotomy mitigated these behavioral anomalies. Thus, *F. rodentium* conversion of resilient Ephx2 KO mice to a depressive phenotype implicates the MGB axis modulation. The importance of regulating the subdiaphragmatic vagus nerve system has been demonstrated for facilitating communication between the gastrointestinal microbiota and the brain. Pu et al. investigated that Chrna7 KO mice fecal microbiota induced depression-like phenotypes in ABX-treated mice, characterized by systemic inflammation and downregulation of PFC synaptic proteins (71). Subphrenic vagotomy performed on mice after FMT significantly prevented the development of the depressive-like phenotype. FMT from Chrna7 KO mice induced depression in ABX mice by modulating the subphrenic vagus nerve, underscoring the potential involvement of the brain-gut microbiome axis in depression development via the vagus nerve.

**Figure 2:**
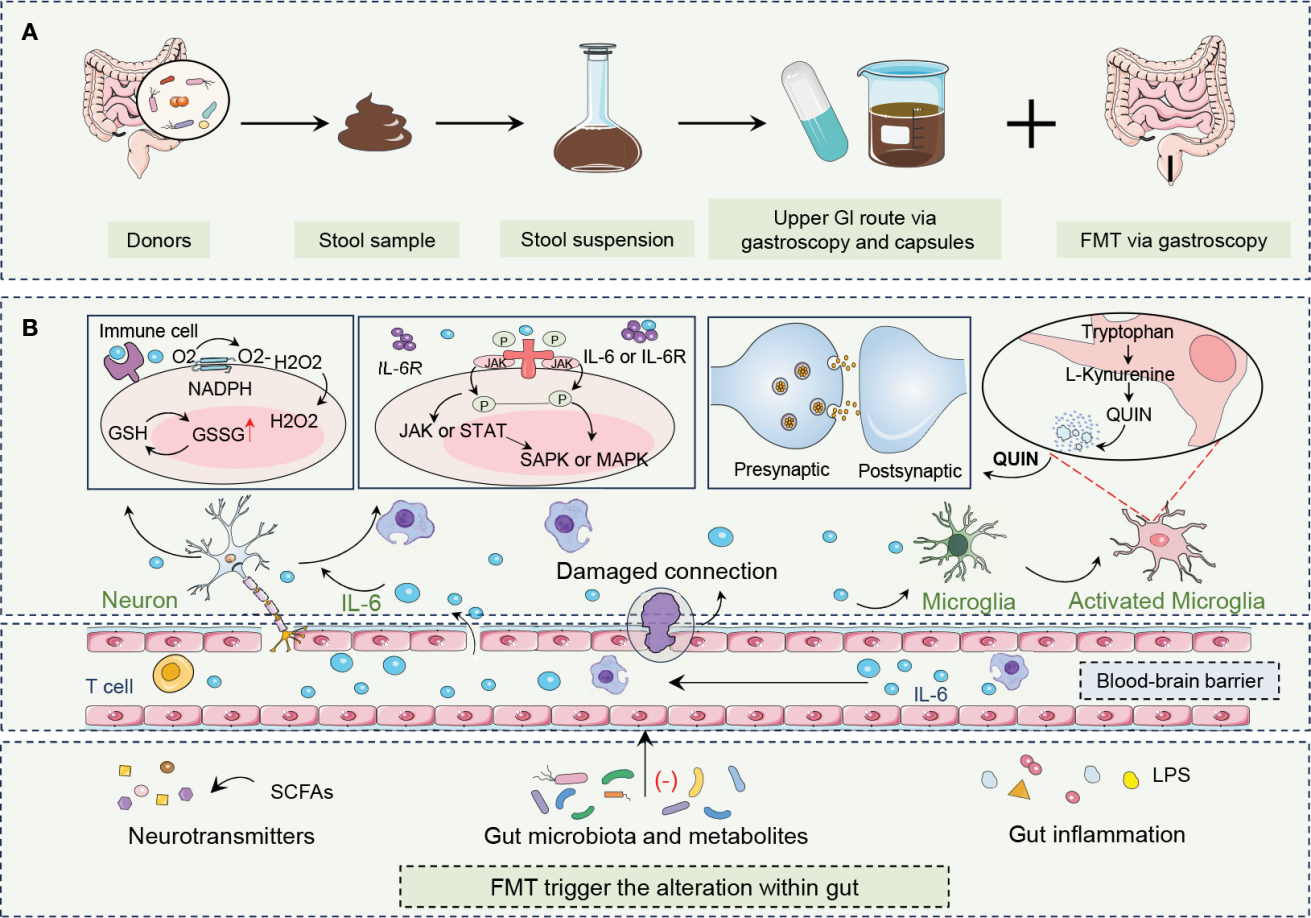
FMT procedure and its roles in combating depression. **(A)** FMT is an innovative treatment that encompasses the acquisition, processing, and administration of fecal material from healthy donors to patients with depression. **(B)** By altering neurotransmitters, gut microbiota and metabolites, and gut inflammation, FMT rebalances gut biota enhances microbe-gut-brain axis communication, restores neurotransmitter homeostasis, and reduces neuroinflammation. FMT modulation of the immune system, particularly through the alteration of IL-6 levels, is crucial to alleviating depressive symptoms. FMT can influence the central nervous system by altering gut microbiota composition and activating beneficial signaling pathways, including those mediated by the vagus nerve, which is a major neural link between the gut and the brain. Fecal microbiota transplantation, FMT; Gastrointestinal, GI; Glutathione, GSH; Oxidized glutathione, GSSG; Hydrogen peroxide, H2O2; Interleukin 6, IL-6; Interleukin 6 receptor, IL-6R; Janus kinase, JAK; Lipopolysaccharide, LPS; Mitogen-activated protein kinase, MAPK; Nicotinamide adenine dinucleotide phosphate hydrogen, NADPH; Oxygen, O2; Quinolinic acid,QUIN; Stress-activated protein kinase, SAPK; Short-chain fatty acids, SCFAs; Signal transducer and activator of transcription, STAT.

Future research endeavors are pivotal in exploring the long-term therapeutic efficacy, safety, and stability of the microbial community post-transplantation in patients with depression. Furthermore, the precise identification of specific microbial strains with therapeutic potential for depression, along with the development of more targeted microbial modulation strategies, will advance the application of FMT in the realm of mental health (**Figure 2**).

## 4 Preclinical evidence of FMT in treating depression

### 4.1 Key targets associated with FMT in treating depression

#### 4.1.1 Sigma-1

Sigma receptors are classified into two subtypes, including Sigma-1 and Sigma-2 (72). The Sigma-1 receptor (Sig-1R), a 28 kD molecular chaperone protein, is pivotal in regulating various cellular processes including intracellular calcium homeostasis, apoptosis, and cell membrane permeability (73). Its role in influencing neuronal survival and function has attracted considerable attention as a potential therapeutic target for central nervous system diseases (74). Li et al. discovered that Sig-1R KO mice exhibited depression-like behavior and gut microbiota disorder, while the depressive behavior was improved after the removal of gut microbiota through ABX treatment (75). After FMT of the Sig-1R KO group into recipient mice, the mice exhibited depression-like behavior, along with a significant decrease in the diversity and abundance of the gut microbiota, specifically *Alistipes*, *Alloprevotella*, and *Lleibacterium*. In addition, the cAMP/CREB/BDNF signaling pathway was inhibited, while the expression of CTNF, TGF-α and NGF was decreased. The results revealed that the gut microbiota from Sig-1R KO mice induced depression-like behavior by modulating the cAMP/CREB/BDNF signaling pathway, and furnished supportive evidence for subsequent investigations into the brain-gut axis.

#### 4.1.2 NLRP3

The inflammasome regulates immune and inflammatory responses in the gut axis of the brain, influencing the synthesis and release of neurotransmitters, which in turn affects neural activity and emotional states in the brain (76–78). The NLRP3 inflammasome is a complex comprising Nucleotide-binding and oligomerization domain, Leucine-rich repeat, and Pyrin, and is involved in multiple diseases (79–81). NLRP3 could be located intracellularly in neurons, astrocytes, and microglia (82). Upon activation, it initiates an intracellular signaling cascade aimed at reinstating homeostasis (83). Given the critical role of NLRP3 in gut-immune-brain communication, deciphering the role and dysfunction of NLRP3 is crucial for depression (83). Zhang et al. transplanted the fecal microbiota of NLRP3 KO mice into chronic unpredictable stress (CUS) mice (84). Depression-like behaviors of the mice were significantly improved after FMT, accompanied by alleviated astrocyte dysfunction. In addition, FMT suppressed the elevation of circHIPK2 levels in CUS mice. The gut microbiota of NLRP3 KO mice regulated astrocyte dysfunction via circHIPK2, attenuating depressive-like behaviors and providing a novel strategy for the treatment of depression.

In ABX-treated rats that received FMT of CUMS, Huang et al. discovered that inflammasomes and inflammatory cytokines IL-1β and IL-18 were increased, and tight junction proteins Occludin and ZO-1 were decreased (85). Furthermore, in recipient rats, the relative abundance of *actinobacteria*, *proteobacteria*, *patescibacteria*, *Lactobacillaceae*, and *erysipelotrichichaceae* was highly upregulated while that of *lachnospiraceae* was significantly downregulated. The microbiota composition was partially overlapped with that of donor rats. Collectively, modulating the gut microbiota composition mitigated inflammation and depressive symptoms by reshaping the microbiota and inhibiting NLRP3 inflammasome activation.

### 4.2 FMT in alleviating depression via beneficial herbs

At present, many Chinese herbs play an important role in alleviating depressive symptoms, and their active ingredients can affect neurotransmitter levels, regulate the neuroendocrine system, and improve psychological states (86). Notable herbs like plant polysaccharides, Xiao-Chai-Hu-Tang, fermented red ginseng, and Zhi Zi Chi decoction have been recognized for their antidepressant pharmacological effects.

#### 4.2.1 Plant polysaccharide

Plant polysaccharide (OP) is a kind of OP extracted from plants, especially from Chinese herbs. Common plant OP includes ginkgo biloba OP, okra OP, yellow extract OP, ginseng OP, bupleurum OP, and so on (87). The OP has various biological functions, such as improving immune function, anti-oxidation, anti-virus, and regulating intestinal microecology (88). In recent years, studies have shown that OP from different sources plays an important role in the regulation of gut microbiota, which can significantly affect the occurrence of depression-like behaviors by regulating gut microbiota and related pathways.

Treatment with Ginkgo biloba leaves (GPS) was shown to reverse the reduction in serotonin-positive and dopamine-positive cell density induced by unpredictable chronic mild stress mice, thereby improving depressive-like behavior (89). The antidepressant effects of GPS OP were likely mediated through its regulation of gut microbiota imbalances associated with depression and an increase in lactic acid bacteria abundance, particularly *Lactobacillus reuteri*. The isolated OP from okra (Abelmoschus esculentus (L) Moench) possesses the capability to hinder the activation of the inflammatory response in the colon, serum, hippocampus, and BV2 cells (90). Additionally, OP could regulate dysbiosis in gut microbiota, alterations in short-chain fatty acids, down-regulation of the TLR4/NF-κB pathway, and enhancement of MAPK signaling. Transplantation of OP-modulated microbiota into CUMS receptor mice alleviated depression and anxiety, reduced elevated cytokine levels (TNF-α, IL-1β, IL-6, etc.), and restored histopathological damage in the colon. OP exhibited antidepressant effects through its anti-inflammatory properties and modulation of the gut microbiota. The underlying mechanism of OP for antidepressant-like effects was closely associated with bidirectional communication within the microbiota-gut-brain axis through the regulation of inflammatory responses. Additionally, novel agar-oligosaccharides (NAOs) treatment significantly improved depressive symptoms in chronic restraint stress (CRS) mice, decreasing IL-18 levels in serum, increasing 5-HT levels in serum and brain, and elevating BDNF levels (91). Thus, NAOs exerted an antidepressant effect by raising levels of serotonin and BDNF in the brains of mice and by reorganizing the gut microbiota. FMT from polysaccharide peptide (PSP)-treated mice to CUMS subjects ameliorated depressive behaviors via hypothalamic-pituitary-adrenal (HPA) axis modulation (92). Post-FMT, increased 5-HT, norepinephrine, ZO-1, and occludin, and decreased hippocampal pro-inflammatory cytokines, corticosterone, LPS, and interferon-γ were observed. In summary, PSP administration exerted antidepressant effects through the MGB axis by modulating PI3K/AKT/TLR4/NF-κB and ERK/CREB/BDNF pathways.

#### 4.2.2 Xiao-Chai-Hu-Tang (XCHT)

XCHT, as an effective treatment for depression, is composed of seven herbal extracts bupleurum, Spinelli, Scutellaria, jujube, ginseng, ginger, and licorice (93). Early investigations suggest that XCHT exhibits potential antidepressant properties by modulating immune responses, inhibiting angiogenesis, and inducing apoptosis in tumor cells (94). To examine the impact of XCHT on tumors associated with depression, Shao et al. implemented a xenograft colorectal cancer mouse model exposed to CRS (95). Transplantations of XCHT-regulated microbiota into CRS-associated xenografted mice showed that XCHT treatment regulated the gut microbiota, inhibited activation of the TLR4/MyD88/NF-κB signaling pathway, and regulated inflammatory cytokine levels, resulting in significant anti-tumor effects *in vivo*. Moreover, XCHT partially ameliorated disruptions in the gut microbiota and depressive symptoms in cancer patients by reducing the abundance of bacteria in the families *Parabacteroides*, *Blautia*, and *Ruminococcaceae* bacterium. As a result, XCHT exerted antitumor activity by inhibiting the TLR4/MyD88/NF-κB signaling pathway through the regulation of the gut microbiota. Gut microbiota might be potentially a novel target for XCHT in the treatment of comorbid depression in anticancer therapies.

#### 4.2.3 Fermented red ginseng

Fermented red ginseng (fRG) undergoes processing that alters its chemical composition, potentially augmenting the concentration of active compounds and yielding novel bioactive metabolites (96) (97). In contrast to conventional red ginseng (RG), fRG exhibits enhanced pharmacological properties, offering promising prospects for augmenting immune function, enhancing energy levels, ameliorating cognitive function, and fostering overall health and well-being. FRG has been shown to mitigate hippocampal neuronal damage in mice and modulate the function of the HPA axis, thereby exerting an antidepressant effect. Shin et al. created mouse models of anxiety/depression (AD) and colitis by subjecting them to chronic immobilization stress or FMT from individuals with ulcerative colitis and depression (98). Oral administration of fRG or RG attenuated hippocampal and hypothalamic expression, and serum corticosterone levels induced by unpredictable chronic mild stress. Similarly, oral ingestion of fRG, RG, ginsenoside Rd, or compound K mitigated stress-induced AD-like behaviors, circulating IL-6 and corticosterone, colonic IL-6 and TNF-α levels, and dysbiosis of the gut microbiota.

#### 4.2.4 Zhi Zi Chi decoction (ZZCD)

Additionally, ZZCD, consisting of *Gardenia jasminoides J. Ellis* and *Glycine max (L.) Merr* is also extensively utilized for addressing anxiety and depression (99). Tian et al. used corticosterone combined with chronic constraint stress to establish the model of anxiety and depression, and transplanted fecal intestinal flora of the ZZCD group into anxious and depressed mice (100). ZZCD exerted an influence on and participated in the neuroactive ligand/receptor interaction process, regulated the HPA axis, influenced the secretion of prolactin and estrogen, interfered with MAPK and TNF signaling pathways, and reduced inflammation levels, thus contributing to inhibiting anxiety and depression.

## 5 Clinical evidence of FMT in treating depression

### 5.1 FMT in improving depressive behavior in patients with IBS

Patients with irritable bowel syndrome (IBS) increasingly exhibit a wide range of neuropsychiatric symptoms, such as worsening gastrointestinal physiology, including visceral hypersensitivity, altered intestinal membrane permeability, and gastrointestinal motor dysfunction (101). In a clinical trial, 18 IBS patients with mild to modest anxiety and depression were recruited and then divided into FMT treatment and control groups (102). FMT effectively alleviated anxiety, depression, and IBS symptoms, with significant improvements in the quality of life. Decreased levels of isovaleric and valeric acids were observed in the FMT group, along with a reduced abundance of specific bacteria. Key pathways affected by FMT were identified, and *Bifidobacterium* and *Escherichia* were highlighted as pivotal in IBS-D pathogenesis and recovery. This study underscored the therapeutic potential of FMT for IBS patients with anxiety and depression. Guo et al. conducted a randomized controlled trial investigating FMT in IBS-D patients with diarrhea and symptoms of anxiety and depression (103). Post-treatment, these patients showed significant improvements in IBS symptoms, stool consistency, and reductions in anxiety and depression scores. FMT therapy enhanced gut microbiota diversity, particularly increasing *Bacteroidetes* and *Firmicutes* abundance, and helped restore microbial balance. This suggests FMT potential in treating IBS-D with co-occurring psychological symptoms.

### 5.2 FMT in improving primary depression

FMT has emerged as a promising treatment in animal models, prompting researchers to explore its potential application in managing depression in human patients. Green et al. conducted a randomized controlled trial in which eligible adult patients with MDD were selected and treated with enema FMT and placebo (104). The study revealed the absence of serious or severe adverse events in either treatment group, along with no significant disparity in mild to moderate adverse events between the experimental and control cohorts. Moreover, the active FMT cohort exhibited notable enhancements in mean gastrointestinal symptom scores, as assessed by the Gastrointestinal Symptom Rating Scale, compared to the placebo cohort. The active FMT group demonstrated superior improvements in quality-of-life measures. These demonstrated that enema-administered FMT was safe and acceptable as an adjunctive treatment for adults with MDD, and improved gastrointestinal symptoms and quality of life to some extent, supporting the association of IBS with a high co-morbidity of MDD.

## 6 Discussion

FMT has emerged as a versatile intervention for a broad spectrum of diseases (105). Beyond its effectiveness in gastrointestinal conditions such as chronic constipation, diarrhea, IBS, inflammatory bowel disease, and other functional intestinal disorders, FMT is increasingly recognized for its potential in neuropsychiatric conditions, including autism spectrum disorder, anxiety disorders, and Parkinson’s disease (106, 107). Significantly, FMT exhibits particular potential as a therapeutic avenue for depression. By influencing the gut-brain axis, which encompasses neural, endocrine, and immune interactions, FMT can modulate mood and behavior (68). This is achieved through the alteration of the gut microbiota composition, evidencing distinct differences in microbial profiles between healthy individuals and those suffering from depression (**Table 1**).

**Table 1:** Evidence of FMT in treating depression and related disorders.

Feces of Donors	Recipients	Function and mechanism	Type of Study	Ref.
Chronic social defeat stress-susceptible mice	ABX-treated Ephx2 Knockout (KO) mice	Triggered depressive-like behaviors by the microbial-gut-brain (MGB) axis modulation	Experimental study	(70)
Chrna7 KO mice	Antibiotics (ABX)-treated mice	Induced depression in ABX mice by modulating the subphrenic vagus nerve	Experimental study	(71)
Sigma-1 receptor (Sig-1R KO) mice	Recipient mice (wild-type mice)	Induced depression-like behavior by modulating the cAMP/CREB/BDNF signaling pathway	Experimental study	(75)
NLRP3 KO mice	Chronic unpredictable stress (CUS) mice	Regulated astrocyte dysfunction via circHIPK2, attenuating depressive-like behaviors	Experimental study	(84)
Chronic unpredictable mild stress (CUMS) rats	ABX-treated rats	Mitigated inflammation and depressive symptoms by reshaping the microbiota and inhibiting NLRP3 inflammasome activation	Experimental study	(85)
Mice treated with Ginkgo biloba leaves polysaccharide (GPS)	Unpredictable chronic mild stress mice	Regulated gut microbiota imbalances associated with depression and an increase in lactic acid bacteria abundance	Experimental study	(89)
Mice treated with the polysaccharide isolated from okra.	CUMS receptor mice	Exhibited antidepressant effects through its anti-inflammatory properties and modulation of the gut microbiota	Experimental study	(90)
Mice treated with Neoagaro-oligosaccharides (NAOs)	Chronic restraint stress (CRS) mice	Reversed the CRS-induced mouse model of depression through modulation of gut microbiota and SCFAs, as well as regulation of 5-HT and BDNF levels	Experimental study	(91)
Mice treated with Polysaccharide peptide (PSP)	CUMS mice	Exerted antidepressant effects through the MGB axis by modulating PI3K/AKT/TLR4/NF-κB and ERK/CREB/BDNF pathways	Experimental study	(92)
Mice treated with Xiao-Chai-Hu-Tang (XCHT)	CRS mice	Exerted antitumor activity by inhibiting the TLR4/MyD88/NF-κB signaling pathway through the regulation of the gut microbiota	Experimental study	(95)
Individuals with ulcerative colitis and depression	Mouse models of anxiety/depression (AD) and colitis	Mitigated stress-induced AD-like behaviors, circulating IL-6 and corticosterone, colonic IL-6 and TNF-α levels, and dysbiosis of the gut microbiota	Experimental study	(98)
Mice treated with Zhi Zi Chi decoction (ZZCD)	Anxious and depressed mice	Regulated the HPA axis, influenced the secretion of prolactin and estrogen, interfered with MAPK and TNF signaling pathways, and reduced inflammation levels, thus contributing to inhibiting anxiety and depression	Experimental study	(100)
Healthy individuals	Irritable bowel syndrome (IBS) patients with mild to modest anxiety and depression	Alleviated anxiety, depression, and IBS symptoms, resulting in significant improvements in the quality of life of the patients	Randomized controlled trial	(102)
Healthy individuals	IBS with diarrhea patients with symptoms of anxiety and depression	Enhanced gut microbiota diversity, helped restore microbial balance, and resulted in improvements in IBS symptoms, stool consistency, as well as reductions in anxiety and depression scores post-treatment	Randomized controlled trial	(103)
Healthy individuals	Adult patients with Major Depressive Disorder (MDD)	Exhibited notable enhancements in mean gastrointestinal symptom scores and demonstrated superior improvements in quality-of-life measures	Randomized controlled trial	(104)

Antibiotics, ABX; Anxiety/depression, AD; Chronic restraint stress. CRS; Chronic social defeat stress, CSDS; Chronic unpredictable mild stress, CUMS; Chronic unpredictable stress, CUS; Ginkgo biloba leaves, GPS; Irritable bowel syndrome, IBS; Knockout, KO; Major depressive disorder, MDD; Microbial-gut-brain, MGB; Neoagaro-oligosaccharides, NAOs; Polysaccharide peptide, PSP; Sigma-1 receptor, Sig-1R KO; Xiao-Chai-Hu-Tang, XCHT; Zhi Zi Chi decoction, ZZCD.

The nervous system is involved in the occurrence, development, and regulation of many diseases (108, 109, 61). The interaction between the microbiome and the central nervous system occurs primarily through the vagus nerve, with afferent fibers transmitting information influenced by microbiome metabolites back to the central nervous system (110). Such interaction is proposed to induce alterations in both central and peripheral systems, potentially alleviating depressive symptoms. Besides, individuals suffering from depression exhibit alterations in both their microbial composition and neurotransmitter levels, disrupting the equilibrium of the gut microenvironment (111). This disruption adversely affects the functionality of the intestinal epithelium, resulting in the dysregulation of the intestinal barrier and the onset of inflammatory responses. Consequently, the compromised intestinal barrier facilitates the passage of intestinal metabolites, microbial components, and even microbial populations, exacerbating systemic inflammatory responses, including imbalances in Th17/Treg cell populations, elevated levels of IL-6, IL-1β, and TNF-α (112). FMT holds promise as a therapeutic intervention by modulating the gut microbiota and stimulating the synthesis of neurotransmitters or their precursors, such as serotonin, dopamine, and GABA, thus potentially ameliorating symptoms associated with depression. These attributes position FMT favorably for both preclinical and clinical depression treatment, offering enhanced adaptability over traditional modalities, thereby significantly improving treatment safety and efficacy (113). The potential for utilizing intestinal flora-based therapies as a fundamental approach to depression treatment holds promising prospects and is anticipated to emerge as a routine and viable alternative. Nonetheless, the practical implementation of FMT encounters numerous challenges due to the influence of various confounding factors on the treatment process.

Firstly, in the field of FMT for treating depression, some critical points remain under-explored. The specific roles of microbial metabolites in the gut-brain axis and their impact on brain function and mood are not fully understood, indicating a need for more targeted research into which metabolites are involved and how they exert their effects (114). Additionally, the function of the gut barrier in depression and how FMT might influence its restoration and relationship with depressive symptoms require further investigation. Moreover, the interplay between the host genetic background and the microbiome, particularly how genetic factors might affect the efficacy of FMT and its influence on specific microbial communities concerning depression, represents a relatively new and promising research area that has yet to be fully delved into.

Secondly, In the context of treating depression, although FMT is an emerging therapeutic approach, with innovation and progress, is currently challenged by the lack of standardized assessment protocols (115). This lack has resulted in the number of bacterial species detected in recipient fecal samples being drastically dependent on the depth of sequencing technology, highlighting inconsistencies in research methodology and the need for further analytical techniques (116). Although the majority of current studies have focused on analyzing changes in fecal microbial composition before and after FMT, there is a distinct lack of research into the detailed description of pathogens and beneficial bacteria associated with depression and the mechanisms by which they interact. In addition, the safety and potential ethical issues of FMT technology cannot be ignored during the transition phase from clinical trials to clinical applications (117). Safety assessment needs to take into account genetic differences between donors and recipients and potential biological risks associated with fecal transplants, which requires an assessment of the patient’s dietary habits, genetic characteristics, and the compatibility of the microbial composition of the donor and the recipient (118). Refined classification and metabolic analyses will provide better information to support clinical decision-making. In addition, the FMT procedure involves human samples and needs to follow compliant medical extraction procedures involving informed consent, privacy rights, and strict moral and medical ethical standards (119–121). Therefore, technical deficiencies in the application of FMT technology in the treatment of depression are mainly due to the lack of a standardized assessment process, insufficient in-depth understanding of the mechanisms of pathogen-beneficial bacterial interactions, and the need for more consideration of safety and ethical issues. These shortcomings emphasize the importance of strengthening technical and methodological research on the application of FMT in the treatment of depression.

Finally, FMT represents a promising yet nascent intervention in the treatment of depression, necessitating rigorous examination of its long-term efficacy and safety due to the inherent complexity and plasticity of the gut microbiome (122). The dearth of definitive clinical trials reinforces a substantial gap in our understanding of FMT capacity to ameliorate depressive symptoms indirectly by modulating gut health, thereby highlighting the imperative for in-depth mechanistic studies (123). Moreover, the prevailing reliance on animal models in the extant literature, coupled with the insufficient validation of clinical trial findings, accentuates the critical need for bridging the translational divide between preclinical insights and clinical application. Such an endeavor warrants prospective investigations to elucidate the nuanced interplay between dysbiosis and depression, aiming to refine our comprehension of the microbial gut-brain axis and the operational mechanism underpinning the therapeutic potential of FMT. Addressing these lacunae will not only pave the way for the establishment of robust clinical protocols but also facilitate the integration of precision medicine approaches, ultimately enabling the development of personalized microbiome-targeting therapies (124).

## 7 Conclusion

FMT offers a cutting-edge approach to depression by modulating the MGB axis, a critical determinant in mental health. By rectifying gut microbiota microenvironment dysbiosis, FMT reinstates a balanced microbial ecosystem, influencing key targets such as the Sig-1R and NLRP3 inflammasome, which are implicated in neuroinflammatory and neurochemical pathways associated with depressive disorders. Additionally, FMT can harness the therapeutic properties of beneficial herbs, further enhancing the antidepressant potential. Despite these promising findings, the complexity of the gut microbiota interaction with the brain and the identification of precise microbial contributors to therapeutic outcomes necessitates advanced research for clinical translation. Standardization of FMT protocols and a deeper understanding of the underlying mechanisms are essential to ensure safety and efficacy in the clinical management of depression.

## Author contributions

QZ: Conceptualization, Data curation, Formal analysis, Methodology, Visualization, Writing – original draft, Funding acquisition, Writing – review & editing. YB: Conceptualization, Visualization, Writing – review & editing, Writing – original draft. BZ: Writing – review & editing, Writing – original draft. QJ: Conceptualization, Writing – review & editing. CM: Writing – review & editing. LL: Writing – review & editing. YD: Writing – review & editing. YL: Writing – review & editing. JY: Conceptualization, Project administration, Supervision, Writing – original draft, Writing – review & editing. WL: Conceptualization, Project administration, Supervision, Writing – original draft, Writing – review & editing. JZ: Conceptualization, Project administration, Supervision, Writing – original draft, Writing – review & editing.

## Glossary

**Table:**

5-FU	5-Fluorouracil
ABX	Antibiotics
AD	Anxiety/depression
BDNF	Brain-derived neurotrophic factor
CEE	Chronic ethanol exposure
CRS	Chronic restraint stress
CUMS	Chronic unpredictable mild stress
CUS	Chronic unpredictable stress
*F. rodentium*	*Faecalibaculum rodentium*
FMT	Fecal microbiota transplantation
fRG	Fermented red ginseng
GABA	Gamma-aminobutyric acid
GPS	Ginkgo biloba leaves
HPA	Hypothalamic-pituitary-adrenal
IBS	Irritable bowel syndrome
KO	Knockout
LPS	Lipopolysaccharide
MDD	Major depressive disorder
METH	Methamphetamine
MGB	Microbial-gut-brain
NAOs	Novel agar-oligosaccharides
OP	Polysaccharide
PSP	Polysaccharide peptide
PFC	Prefrontal cortex
RG	Red ginseng
RA	Rheumatoid arthritis
Sig-1R	Sigma-1 receptor
XCHT	Xiao-Chai-Hu-Tang
ZZCD	Zhi Zi Chi decoction
